# A systematic review assessing the efficacy of doxycycline as adjunct therapy for nodding syndrome

**DOI:** 10.1192/j.eurpsy.2025.1889

**Published:** 2025-08-26

**Authors:** A. A. Kumar, R. Walwaikar, A. A. Pillai, A. Agrawal, F. Sheikh

**Affiliations:** 1 GMERS Medical College, Vadnagar; 2 SSPM Medical College and Lifetime Hospital, Sindhudurg, India; 3 Humanitas University, Milan, Italy; 4 Greater manchester mental health trust, Manchester, United Kingdom

## Abstract

**Introduction:**

The rare epileptic seizure syndrome nodding is endemic among African adolescents. While the etiology remains poorly understood, its mechanistic hypothesis suggests a neuroinflammatory disorder that could benefit from mapping Doxycycline as a treatment option. Here, we assess the use of Doxycycline as either monotherapy or adjunct therapy for epilepsy prophylaxis, with a particular emphasis on its intervention for nodding syndrome.

**Objectives:**

The primary objective of this study is to assess the safety and efficacy of Doxycycline for treating nodding syndrome. Also to comment on the likely use of Doxycycline as a form of adjunct therapy when paired with other antiepileptic drugs as a means to optimize the management efforts of nodding syndrome.

**Methods:**

Our analysis included randomized controlled trials and observational studies which were sorted and assessed in accordance to PRISMA guidelines through a systematic search of the literature using all electronic databases, including PubMed, Google Scholar, Scopus, and Cochrane. The search terms included Doxycycline and nodding syndrome. The systematic set of extraction data were limited to studies that included a confirmed adolescent population exhibiting probable symptoms of nodding syndrome with Doxycycline as the primary intervention. Effect sizes will be measured with a random-effects model, and heterogeneity will be calculated with I² statistics.

**Results:**

Nine studies in total involving 1,120 subjects were analyzed, that included four randomized controlled trials (RCTs) assessing the effects of doxycycline monotherapy on 480 subjects, as well as five observational studies exploring the use of doxycycline with first-line anti-epileptic drugs (AEDs) on 640 subjects.

Seizure frequency reduction

Doxycycline administration on its own reduced seizure frequency by 30% relative to the placebo (relative risk [RR] = 0.70, 95% confidence interval [CI]= 0.60 to 0.85, p < .001), whereas, added to AEDs, reduced seizure frequency by 45% (RR = 0.552, 95% CI = = 0.42 to 0.73, p < 0.001; I² = 22%, considered low heterogeneity).

Lower severity of symptoms

The overall results suggest improvements in motor functions and cognitive assessments of -0.82 (standardized mean difference, 95% CI = -1.12 to -0.52, p < .001), which may indicate an improvement in motor symptoms.

AED add-on and impact on quality of life

Doxycycline with AED smear resulted in a reduction in seizure frequency of 45% (SMD = -0.68, 95% CI = -0.94 to -0.42, p < .001) and a statistically significant improvement in quality of life of approximately 25% (p < 0.01); effect estimates presented moderate heterogeneity (I² = 45%).

**Conclusions:**

Doxycycline has potential for extended use since our findings support a safe and potentially beneficial intervention in nodding syndrome. Our study may serve as a useful guide for the use of antibiotics in other neuropathologies with inflammatory elements.

**Disclosure of Interest:**

None Declared

